# Fixel-Based Analysis and Free Water Corrected DTI Evaluation of HIV-Associated Neurocognitive Disorders

**DOI:** 10.3389/fneur.2021.725059

**Published:** 2021-11-04

**Authors:** Alan Finkelstein, Abrar Faiyaz, Miriam T. Weber, Xing Qiu, Md Nasir Uddin, Jianhui Zhong, Giovanni Schifitto

**Affiliations:** ^1^Department of Biomedical Engineering, University of Rochester, Rochester, NY, United States; ^2^Department of Electrical and Computer Engineering, University of Rochester, Rochester, NY, United States; ^3^Department of Neurology, University of Rochester, Rochester, NY, United States; ^4^Department of Biostatistics and Computational Biology, University of Rochester, Rochester, NY, United States; ^5^Department of Physics and Astronomy, University of Rochester, Rochester, NY, United States; ^6^Department of Imaging Sciences, University of Rochester, Rochester, NY, United States

**Keywords:** HIV, diffusion MRI (dMRI), fixel-based analysis, free water imaging, machine learning, cognitive impairment, brain, white matter (WM)

## Abstract

**Background:** White matter (WM) damage is a consistent finding in HIV-infected (HIV+) individuals. Previous studies have evaluated WM fiber tract-specific brain regions in HIV-associated neurocognitive disorders (HAND) using diffusion tensor imaging (DTI). However, DTI might lack an accurate biological interpretation, and the technique suffers from several limitations. Fixel-based analysis (FBA) and free water corrected DTI (fwcDTI) have recently emerged as useful techniques to quantify abnormalities in WM. Here, we sought to evaluate FBA and fwcDTI metrics between HIV+ and healthy controls (HIV−) individuals. Using machine learning classifiers, we compared the specificity of both FBA and fwcDTI metrics in their ability to distinguish between individuals with and without cognitive impairment in HIV+ individuals.

**Methods:** Forty-two HIV+ and 52 HIV– participants underwent MRI exam, clinical, and neuropsychological assessments. FBA metrics included fiber density (FD), fiber bundle cross section (FC), and fiber density and cross section (FDC). We also obtained fwcDTI metrics such as fractional anisotropy (FA_T_) and mean diffusivity (MD_T_). Tract-based spatial statistics (TBSS) was performed on FA_T_ and MD_T_. We evaluated the correlations between MRI metrics with cognitive performance and blood markers, such as neurofilament light chain (NfL), and Tau protein. Four different binary classifiers were used to show the specificity of the MRI metrics for classifying cognitive impairment in HIV+ individuals.

**Results:** Whole-brain FBA showed significant reductions (up to 15%) in various fiber bundles, specifically the cerebral peduncle, posterior limb of internal capsule, middle cerebellar peduncle, and superior corona radiata. TBSS of fwcDTI metrics revealed decreased FA_T_ in HIV+ individuals compared to HIV– individuals in areas consistent with those observed in FBA, but these were not significant. Machine learning classifiers were consistently better able to distinguish between cognitively normal patients and those with cognitive impairment when using fixel-based metrics as input features as compared to fwcDTI metrics.

**Conclusion:** Our findings lend support that FBA may serve as a potential *in vivo* biomarker for evaluating and monitoring axonal degeneration in HIV+ patients at risk for neurocognitive impairment.

## Introduction

Combined antiretroviral therapy (cART) has reduced morbidity and mortality rates significantly in HIV infected (HIV+) individuals (1). However, the increased survival may be masking an increase in cognitive impairment (2), mediated by injury to the central nervous system (CNS) and disruption of the blood–brain barrier (BBB) (3). The HIV reservoir in the CNS resides primarily in microglia and perivascular macrophages, resulting in chronic neuroinflammation (4). While the small pool of infected cells in the CNS can release neurotoxic viral proteins, Tat and gp120, the larger pool of activated glia cells is responsible for the release of cytokines that can induce neuronal injury and cell death (5). HIV-associated oligodendrocyte injury results in demyelination and alterations in white matter (WM) structural integrity (6). Thus, damage to WM fibers is likely a key factor in cognitive impairment observed in HIV-associated neurocognitive disorder (HAND) (7).

MR neuroimaging studies have sought to identify potential *in vivo* biomarkers to investigate CNS injury in the setting of HIV infection (8). Structural and functional MRI have helped elucidate how atrophy and aberrant network topology mediate cognitive decline in HIV infection (9, 10). Nonetheless, given the well-established presence of WM alterations in HIV infection, it is paramount to further characterize WM in HAND. However, during chronic neuroinflammation, there may be contributing vasogenic edema (11), confounding the interpretation of WM lesions. Accordingly, appropriate models that accurately account for free water (FW) contamination are necessary to sufficiently evaluate WM structural integrity in HIV infection (12).

Due to its non-invasiveness, diffusion tensor imaging (DTI) has been widely used in clinical neuroimaging studies (13, 14). DTI metrics such as fractional anisotropy (FA), axial diffusivity (AD), radial diffusivity (RD), and mean diffusivity (MD) characterize the orientation and distribution of the random movements of water molecules, diffusion magnitude, diffusional directionality perpendicular to the axon, and diffusional directionality along the axon, respectively (15, 16). Previous studies have shown that FA is decreased in the posterior limb of internal capsule (PLIC), the corticospinal tract (CST), and temporal and frontoparietal WM regions, whereas RD and MD were increased in bilateral CST and temporal and frontal WM regions in HIV+ individuals (17–22). Decreased FA has also been observed in the superior longitudinal fasciculus (SLF) and was correlated with decreased memory and executive function in HIV+ subjects exhibiting HAND (23). Tract-based spatial statistics (TBSS) is a popular voxel-based method to analyze DTI metrics, which maps control and disease cohort FA images to a WM skeleton to improve correspondence between subjects (24). We have previously reported diffuse FA and MD abnormalities using TBSS in HIV+ individuals (25).

However, voxel-based measures are often contaminated by extracellular FW (12). FW contamination in the diffusion signal is due to water molecules that are not restricted by their environment, such as the cerebrospinal fluid (CSF). Edema caused by stroke (26), brain tumors (12), or neuroinflammation can also contaminate WM voxels (27, 28). Accordingly, FW contaminated voxels will fit more toward an isotropic tensor and exhibit decreased FA values, confounding the interpretation of the results. Previously, several studies have reported that FW correction enhances specificity of DTI metrics (29–32) and reduces test–retest reproducibility errors (33). However, the diffusion tensor model is limited in that it is not able to reliably model complex and crossing-fiber populations, which are present in up to 90% of WM voxels (34, 35). Furthermore, while TBSS of FW corrected FA (FA_T_) is likely to provide more reliable measures of WM integrity in HIV infection, it does not include orientation information.

Fixel-based analysis (FBA) is a recent technique that models individual fibers at the sub-voxel level, termed fixels, which allow tract-specific comparisons (36). FBA enables the characterization of multiple fiber populations within a voxel, circumventing interpretation issues that commonly arise with voxel-averaged measurements such as FA and MD. Moreover, FBA accounts for both macrostructural (fiber bundle) and microstructural (within voxel) changes within WM, providing a more comprehensive understanding of intra-axonal and fiber tract changes. Accordingly, FBA has been used in several neurological disorders including Parkinson's disease (37, 38), multiple sclerosis (39, 40), traumatic brain injury (41, 42), schizophrenia (42), and healthy aging (43). FBA can be used to estimate fiber density (FD) within a fiber bundle, the fiber bundle cross section (FC), or a combined measure, fiber density cross section (FDC). FD is related to the intra-axonal volume, and a corresponding decrease in FD may reflect axonal degeneration (36). However, correspondence between the apparent fiber density (AFD) and simulated intra-axonal signal fraction is improved at higher *b*-values (44). Accordingly, it is possible that at moderate *b*-values (*b* <2,000 s/mm^2^), the FOD is no longer purely representative of the intra-axonal volume (44). At moderate *b*-values, the DW signal contains contributions from both the intra-axonal and extra-axonal spaces, and the FD needs to be interpreted cautiously. FD or AFD is calculated from the fiber orientation distribution (FOD), which may be estimated using constrained spherical deconvolution (CSD) (45, 46), or advanced multi-tissue constrained spherical deconvolution (MT-CSD) (47), which considers different brain tissue types. By modeling gray matter (GM), WM, and CSF separately, MT-CSD accounts for FW contamination and has been shown to have better test–retest reliability than traditional DTI metrics (48). FC reflects the cross-sectional area of a fiber bundle, perpendicular to the length axis, and is derived from the Jacobian of the non-linear transformation from subject space to template space. By accounting for the orientation of the fiber bundle, FC is reflective of the number of axons within the fiber bundle, and therefore the ability to relay information (36). FDC accounts for both macroscopic and microscopic effects on fiber density.

The aim of this study was to refine our understanding of how WM structural integrity is affected in HIV-infected individuals, and if these changes were associated with cognitive performance in HAND, using two approaches, FBA and fwcDTI. Additionally, we investigated whether fiber tract degeneration was related to inflammatory blood markers NfL and Tau of HIV infection. Machine learning classification, using a set of binary classifiers was also performed to distinguish cognitively normal individuals from those with cognitive impairment in HIV+ individuals.

## Materials and Methods

### Study Participants

Forty-two treatment-naïve HIV+ participants (2 females and 40 males; mean age ± standard error, SE = 34.48 ± 1.95 years, range 20–63 years) and 52 age-matched HIV uninfected (HIV–) participants (26 females and 26 males; mean age ± SE = 37.02 ± 1.66 years, range 18–63 years) were enrolled in a study assessing the potential neurotoxicity of combination antiretroviral therapy treatment (cART) study at the University of Rochester Medical Center. All participants provided written informed consent before enrollment according to the institutional protocol and underwent clinical, laboratory, and brain MRI exams. All experiments were performed in accordance with relevant guidelines and regulations. The data reported here reflect the baseline assessment of HIV+, cART naïve individuals prior to starting cART. This time point was chosen because it represents the clearest difference in cognitive performance within the HIV+ group. Details about study participants (including age, sex, and clinical results) are provided in Table 1.

**Table 1:** Participant demographics, clinical, and cognitive information.

		**HIV+ (*N* = 42)**	**HIV– (*N* = 52)**	***p*-value**
Age, mean(SE)		34.05 (2.01)	37.13 (1.65)	0.24
Gender, *n* (%)				
Female		2 (0.048)	26 (0.5)	<**0.001**
Male		40 (0.952)	26 (0.5)	
Ethnicity, *n* (%)				
Hispanic or Latino		2 (0.048)	3 (0.058)	0.829
Not Hispanic or Latino		40 (0.952)	49 (0.942)	
Race, *n* (%)				
Caucasian		20 (0.476)	43 (0.827)	
Black		21 (0.5)	5 (0.096)	<**0.001**
Other		1 (0.024)	2 (0.038)	
Missing		0 (0)	1 (0.019)	
Education, *n* (%)				
<12 years		2 (0.048)	2 (0.038)	
12 years (HS grad)		11 (0.26)	4 (0.077)	
12–15 years (Some college)		16 (0.38)	10 (0.19)	**0.002**
16 years (Bachelor's)		7 (0.17)	21 (0.40)	
>16 years (Post-graduate)		6 (0.14)	15 (15.52)	
Blood markers, mean (SE)				
CD4		503.64 (41.99)	–	–
Viral load (×10^3^)		771.22 (205.58)	–	–
Neurofilament (NfL)		11.39 (1.91)	–	–
Tau		2.47 (1.07)	–	–
Cognitive score				
Total summary score		−1.45 (0.386)	0.325 (0.444)	**0.035**
Executive *z*-score		−0.122 (0.083)	0.227 (0.108)	>0.05
Attention *z*-score		−0.294 (0.101)	0.130 (0.092)	**0.033**
Motor *z*-score		−0.208 (0.097)	0.246 (0.086)	**0.016**
Learning *z*-score		−0.309 (0.082)	−0.153 (0.100)	>0.05
Speed of learning *z*-score		−0.188 (0.091)	0.042 (0.096)	>0.05
Memory *z*-score		−0.261 (0.096)	−0.223 (0.102)	>0.05
Verbal *z*-score		−0.073 (0.086)	0.057 (0.106)	>0.05
HAND classification *n* (%)				
WNL		21 (50%)	NA	NA
CI	ANI	20 (48%)		
	MND	1 (2%)		

*Continuous variables summarized as Mean (SE), categorical variables are summarized as frequency (percentages). CD4 in cells/mm^3^, NfL in pg/ml. Significant results are shown as bold. SE, standard error; HAND, HIV-associated neurocognitive disorders; WNL, within normal limits; CI, cognitive impairment; ANI, asymptomatic neurocognitive impairment; MND, mild neurocognitive disorder; NA, not applicable*.

### Data Acquisition

#### Blood Sample

Plasma levels of markers associated with neuroinflammation and neurodegeneration (Neurofilament light chain NfL, and Tau protein) were measured by Simoa assay via commercial lab, Quanterix^TM^ (Lexington, MA, United States, https://www.quanterix.com/). Viral load (VL) from each HIV+ participant was measured via Roche COBAS 8800 System with a lower limit of detection of 20 copies/ml. CD4+ count was obtained via flow cytometric immunophenotyping at the Clinical Laboratory Improvement Amendments (CLIA), certified clinical lab at the University of Rochester.

#### Neuropsychological Assessments

The neurocognitive evaluation was performed by trained staff and supervised by a clinical neuropsychologist. Tests of Executive Function (Trailmaking Test Parts A & B, Stroop Interference task), Speed of Information Processing (Symbol Digit Modalities Test and Stroop 2 Color Naming), Attention and Working Memory [CalCAP(CRT4) and WAIS-III Letter-Number Sequencing], Learning [Rey Auditory Verbal Learning Test AVLT (trials 1–5), Rey Complex Figure Test Immediate Recall], Memory (Rey Auditory Verbal Learning Test RAVLT Delayed Recall, Rey Complex Figure Test Delayed Recall), and Motor (Grooved Pegboard, left and right hand) were administered at each visit. Premorbid intellectual functioning ability was estimated via WRAT-4 Reading at the baseline visit only. Raw scores were converted to *z*-scores using test manual norms but the *z*-scores were cut off at ±3 standard deviations (SD) above and below the mean values. Cognitive domain scores were created by averaging the *z*-scores of tests within each domain. A total summary score was calculated by summing the *z*-scores of the six cognitive domains measured (Executive Function, Speed of Information Processing, Attention and Working Memory, Learning, Memory, and Motor). HAND diagnoses were determined for each participant according to the Frascati criteria (49). Participants were accordingly defined as either within normal limits (WNL) or cognitively impaired (CI) [i.e., participants having asymptomatic neurological impairment (ANI) or minor neurocognitive impairment (MND)]. The ANI was defined as neuropsychological impairment (>1 SD below the demographically appropriate normative mean) in 2 or more cognitive domains with no functional decline (as measured by the Instrumental Activities of Daily Living Scale), while mild neurocognitive disorder (MND) was defined as neuropsychological impairment in two or more cognitive domains with mild functional decline (49).

#### Image Acquisition

All participants were scanned on a 3 T MRI scanner (MAGNETOM Trio, Siemens, Erlangen, Germany) equipped with a 32-channel head coil.

##### Anatomical Imaging

For the purpose of segmentation and identification of the anatomical landmarks, T1-weighted (T1w) images were acquired using a 3D magnetization prepared rapid acquisition gradient-echo (MPRAGE) sequence with Inversion Time (TI) = 1,100 ms, Repetition Time (TR) = 2,530 ms, Echo Time (TE) = 3.44 ms, Flip Angle = 7, Field of View (FOV) = 256 × 256, GRAPPA factor = 2, number of average = 1, number of slices = 192, voxel size = 1.0 × 1.0 × 1.0 mm^3^, and total time of acquisition (TA) was 5 min 52 s.

##### Diffusion Tensor Imaging

Diffusion-weighted images (DWI) were acquired using a single-shot spin echo echo-planar imaging (SE-EPI) sequence with 60 non-collinear diffusion-encoded images (*b* = 1,000 s/mm^2^), 10 non-diffusion-weighted reference images (*b* = 0 s/mm^2^), TR = 8,900 ms, TE = 86 ms, FOV = 256 × 256, GRAPPA factor = 2, number of slices = 70, number of volumes = 61, voxel size = 2.0 × 2.0 × 2.0 mm^3^, TA = 10 min 51 s. In order to correct for EPI distortions, a double-echo gradient echo field map sequence was also acquired (TR = 400 ms; TE = 5.19 ms, FOV = 256 × 256, flip angle = 60, number of slices = 70, voxel size = 2.0 × 2.0 × 2.0 mm^3^, TA = 3 min 28 s).

#### Image Preprocessing

All MRI images were visually inspected for any severe artifacts. DWI images were corrected for eddy current-induced distortion, susceptibility-induced distortion, and motion correction using TOPUP and EDDY tools in FSL [https://fsl.fmrib.ox.ac.uk/fsl/fslwiki/; (50, 51)].

##### Fixel-Based Analysis

FBA was performed using the recommended pipeline in MRtrix3 (www.mrtrix.org, version 3.0.2) (36, 52). Briefly, DWI images were up-sampled by a factor of 2 in all three dimensions using tri-cubic interpolation. The fiber orientation distribution functions (FODs) within each voxel were computed in the following way: response functions for single-fiber WM, GM, and CSF were estimated from single-shell data using an unsupervised method (53). Single-shell three-tissue CSD (SS3T-CSD) was then performed to obtain FODs for WM, GM, and CSF compartments (54) using MRtrix3Tissue (https://3Tissue.github.io), a fork of MRtrix3 (52). A study-specific template was then created by spatial normalization of subjects using symmetric diffeomorphic non-linear transformation FOD-based registration (55). One group-averaged FOD template was created for cross-sectional analysis, including 20 HIV+ and 20 HIV– individuals. The FOD image for each subject was then registered to the template using FOD-guided non-linear registration.

A tractogram was then generated using whole-brain probabilistic tractography on the FOD population template (52). Twenty million streamlines were generated and subsequently filtered to two million streamlines using spherical deconvolution informed filtering of tractograms (SIFT) to reduce reconstruction biases (56). Fixel-specific measures of fiber density (FD) and fiber bundle cross section (FC) were calculated within each voxel. The log of FC (logFC) was calculated to ensure FC values were centered around zero and normally distributed. A combined measure, FDC, was also generated by multiplying FD and FC. FD, logFC, and FDC for all fixels within a given ROI were then averaged, determined using a fixel mask for the major fiber bundles, using the Johns Hopkins University (JHU) DTI-based WM atlas.

##### DTI Preprocessing

DTI metrics (FA and MD) were computed using DTIFIT in FSL (57). Free water corrected DTI (fwcDTI) metrics (FA_T_ and MD_T_) were computed with a bi-tensor model from the DWI using a previously described algorithm (58) and the processing was performed using Nextflow pipeline (59) with all software dependencies bundled in a Singularity Container (60).

### Statistical Analysis

#### Participant Characteristics

Differences in clinical parameters between HIV+ and HIV– cohorts at baseline were examined using two-way independent *t*-tests at the α = 0.05 significance level. Statistical analysis of demographic data was computed in R 3.6.2 (R Foundation for Statistical Computing, Vienna, Austria).

Univariate comparisons between two independent groups were conducted by either two-group Welch's unequal variances *t*-test (for continuous variables) or Fisher exact test (for categorical variables). Pearson correlation test was used to test the univariate associations between two continuous variables. A *p* < 0.05 was considered statistically significant for a single hypothesis testing problem. For inferential problems that involved multiple hypotheses, Benjamini–Hochberg multiple testing procedure was used to control the false discovery rate (FDR) at α < 0.05 level (61).

#### Fixel-Based Analysis

##### Whole-Brain Fixed Based Analysis

Statistical analyses of images were performed in MRtrix3 (www.mrtrix.org, version 3.0.2) (52). All WM fixels were compared between HIV+ and HIV– individuals. Group comparisons were performed for FD, logFC, and FDC at each fixel using a General Linear Model (GLM), with age and sex included as covariates. Connectivity-based fixel enhancement (CFE) and non-parametric permutation testing over 5,000 permutations were used to identify significant differences in fixel-based metrics (62). Family-wise error (FWE)-corrected *p*-values are reported to account for multiple comparisons. Significant fixels (FWE corrected *p* < 0.05) were visualized using the *mrview* tool in MRtrix3. Fixels were mapped to streamlines of the template-derived tractogram, only displaying streamlines corresponding to significant fixels. Significant streamlines were colored by the effect size, presented as a percentage relative to HIV– individuals or by streamline direction (left–right, red; inferior–superior, blue; anterior–posterior, green).

##### Region of Interest Analysis

In addition to whole-brain analysis, region of interest (ROI) analysis was also performed for fixel-based metrics (FD, logFC, and FDC), DTI metrics (FA and MD), and fwcDTI metrics (FA_T_ and MD_T_) along specific WM tracts using the JHU DTI-based WM atlas. ROIs were chosen to reflect canonical WM pathways using previous studies investigating WM changes in HIV infection (20, 21). The following ROIs were included in the analyses: the left and right posterior limb of internal capsule (PLIC), the left and right superior corona radiata (SCR), the left and right cerebellar peduncles (CP), the left and right inferior cerebellar peduncle (ICP), and the middle cerebellar peduncle (MCP). These regions were chosen *a priori* based on the findings from whole-brain FBA. The mean value for FD, logFC, and FDC was computed for each ROI and compared across groups. Correlation analyses were also performed to evaluate the relationship between fixel-based metrics and cognitive *z*-scores. Independent *t*-tests with multiple comparison corrections were used to compare mean logFC, FD, and FDC between cohorts over the ROIs. Correlations were performed using the non-parametric Spearman's rho and a linear model with age and sex as covariates. Benjamini–Hochberg procedure was applied to control the FDR at α < 0.05 significance level.

#### Tract Based Spatial Statistics (DTI and fwcDTI)

FSL-based TBSS was performed to investigate the FA, free water corrected FA (FA_T_), MD, and free water corrected MD (FA_T_) changes along WM tracts (24). Group comparisons were performed using FSL Randomize for 5,000 permutations. Threshold-free cluster enhancement (TFCE) (63) was used for multiple comparison correction at the α = 0.05 significance level.

#### Machine Learning Classification

Machine learning classification was performed using FBA and fwcDTI metrics for HIV+ individuals. Classifiers were implemented in scikit-learn (64). Mean values within an ROI that were determined to be significant between HIV+ and HIV– subjects were used as input, corresponding to 12 input features. Instances were standardized prior to training and dimensionality reduction was performed using kernel principal component analysis (kPCA) to two features. Four binary classifiers were used to evaluate the specificity of both fixel-based metrics and fwcDTI metrics in their ability to distinguish between WNL and CI. In this study, we implemented random forest, naïve Bayes, linear discriminant analysis (LDA), and adaptive boosting (AdaBoost) classifiers. All classifiers were optimized using a grid search algorithm with a stratified five-fold cross-validation. Classifiers were evaluated using the weighted average (across classes) for precision, recall, and f1-score. Precision, also known as the positive predictive value (PPV), is defined as the number of instances classified as positive, divided by the total number of positive (CI) instances. Recall, or sensitivity, is the number of instances accurately classified as positive (true positives), divided by the total number of instances classified as positive. The *F*-score is the harmonic mean of precision and recall. Receiver operating characteristic (ROC) curves and precision-recall curves (PRC) were also evaluated to assess the performance of these classifiers. Given the small dataset, results are reported as the average across five-fold.

## Results

### Participant Characteristics

Clinical characteristics, demographic information, and cognitive scores for HIV+ and HIV– individuals are presented in Table 1. HIV+ and HIV– cohorts did not significantly differ in age or ethnicity. The total summary *z*-score, attention *z*-score, and motor *z*-score were found to be significantly lower in the HIV+ cohort (*p* < 0.05; Supplementary Figure S1).

### Whole-Brain Fixel-Based Analysis

**Figure 1:**
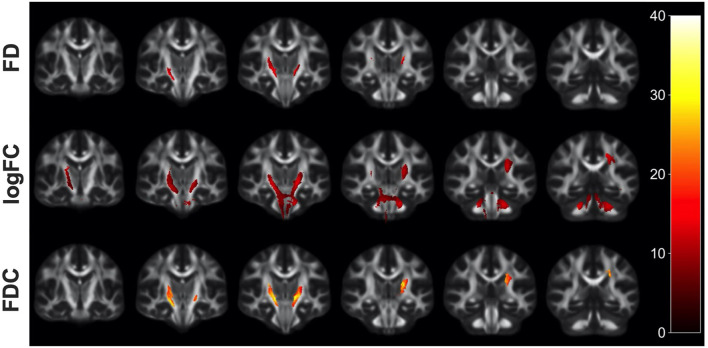
Fiber tract-specific reductions in HIV+ compared to HIV– using whole-brain FBA. Significant fixels (FWE-corrected *p*-value) between HIV+ and HIV– groups displayed as the percentage decrease in the HIV+ group compared to healthy controls, displayed in coronal slices. FD, fiber density; logFC, fiber bundle cross section; FDC, fiber density and cross section.

**Figure 2:**
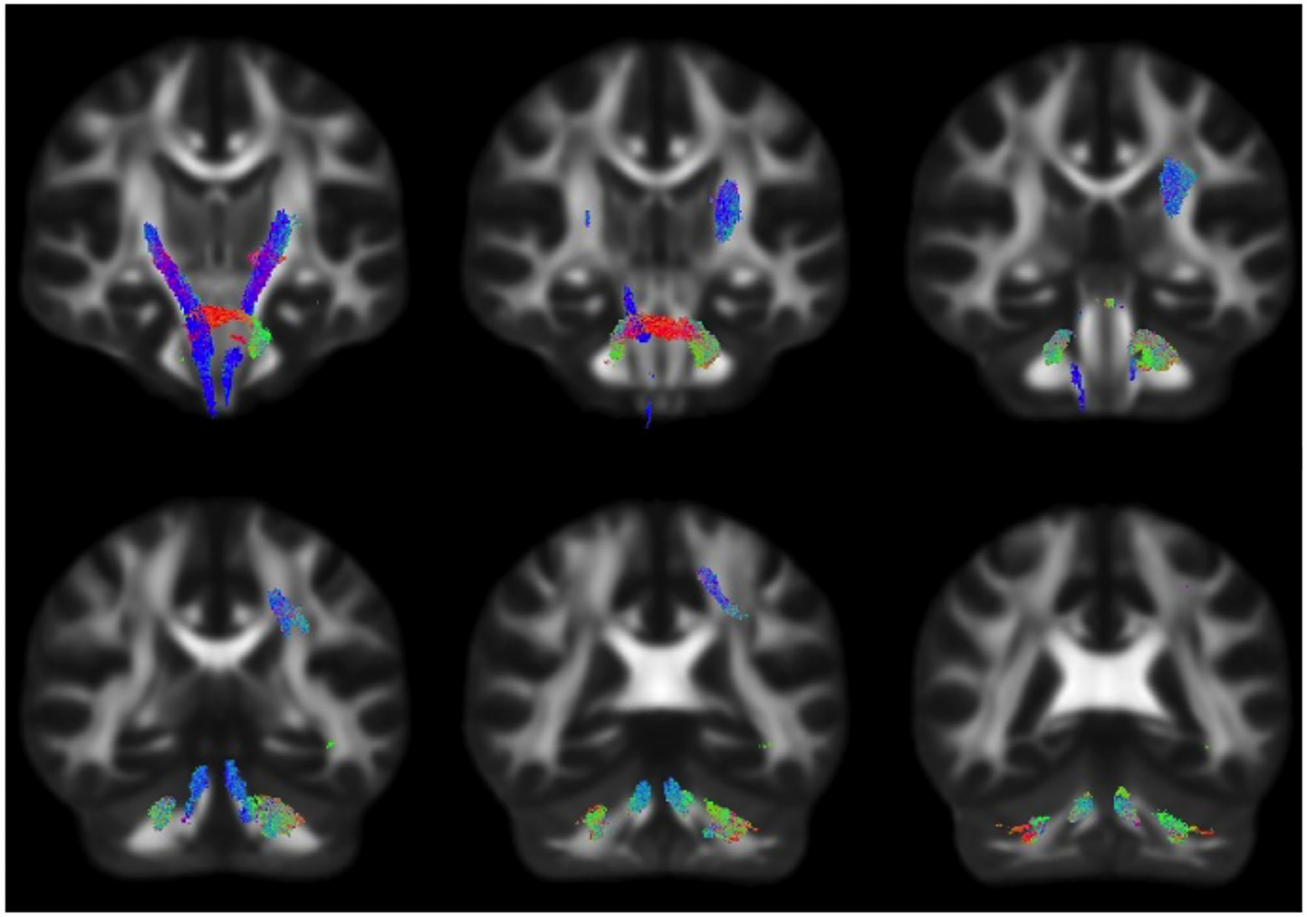
Fiber tract-specific logFC decreases in HIV infection, colored by direction. Streamlines were cropped from the template tractogram to include only significant fixels (FWE-corrected *p* < 0.05) for which the logFC metric is decreased in the HIV+ compared to HIV– individuals. Significant streamlines are shown across coronal slices and colored by direction (anterior–posterior, green; superior–inferior, blue; left–right, red).

**Figure 3:**
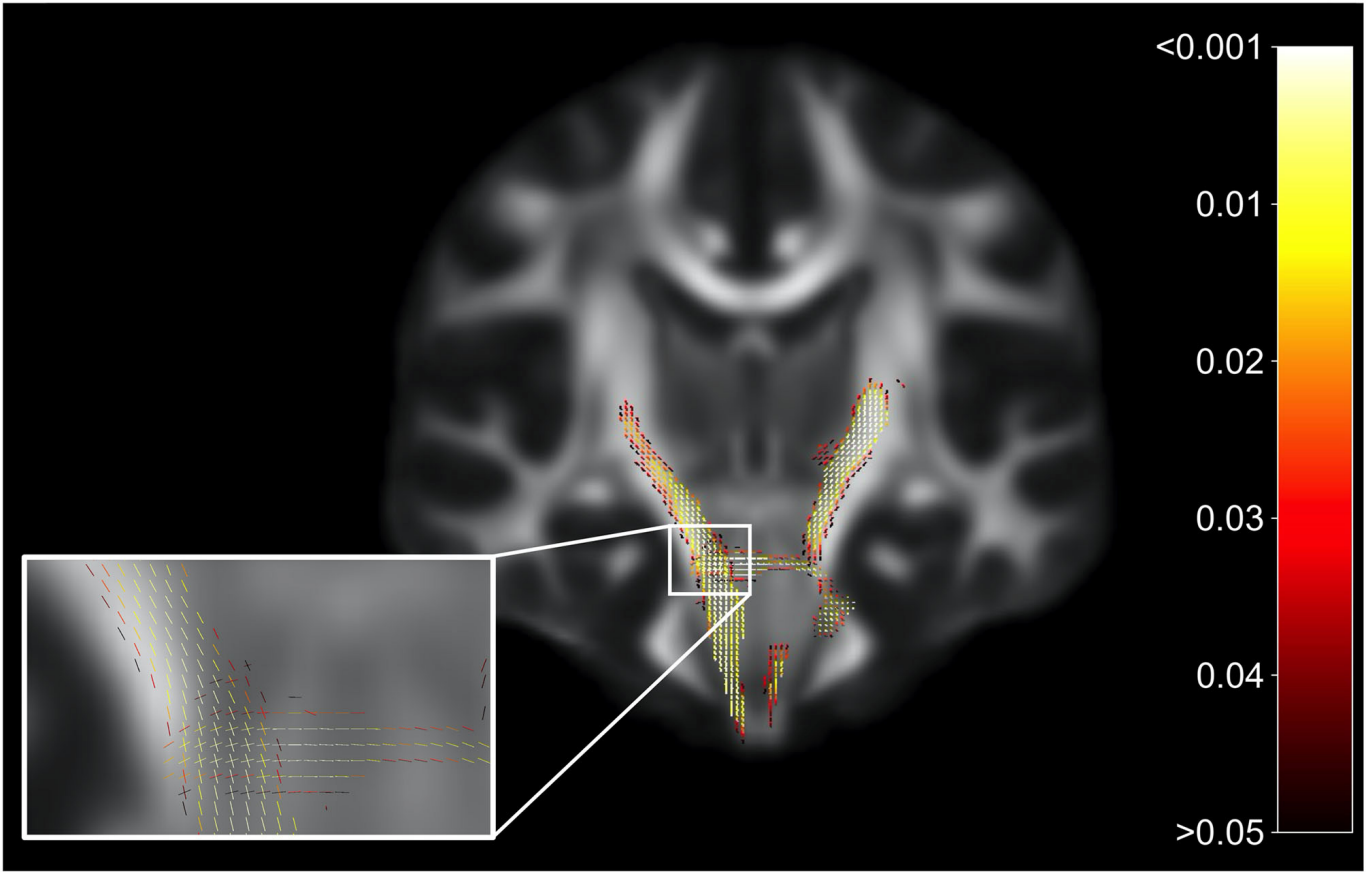
Fiber tract-specific significant fixels. Coronal slice showing fixels that were significantly decreased (FWE-corrected *p*-values) in HIV+ individuals compared to HIV–. The zoomed-in area illustrates differences and *p*-values assigned to individual fixels in regions with crossing-fibers, around the cerebral peduncles (CP) and middle cerebellar peduncles (MCP). Fixels are colored by FWE-corrected *p*-value.

Whole-brain FBA is shown in Figure 1. Streamlines corresponding to significant fixels (FWE corrected *p* < 0.05) are represented as the percentage decrease in HIV+ individuals compared to HIV– individuals for FD, logFC, and FDC. Macrostructural decreases (measured via logFC) of up to 15% were observed along specific fiber tracts. Specifically, the PLIC and MCP were affected bilaterally. The right SCR was also affected. Similar findings were observed for FD, though much less pronounced. Moreover, decreases in FD were more localized to the PLIC. FDC exhibited similar patterns of micro- and macro-structural degeneration, with a larger effect size (Table 2). Compared to HIV– individuals, HIV+ individuals had a 35% decrease in FDC in the PLIC bilaterally as well as the right SCR. Figure 2 shows streamlines displayed and colored based on orientation for significant decreases in logFC in HIV+ individuals. Figure 3 shows a coronal view of fiber tract-specific significant fixels, and the inset shows a zoomed-in area indicating regions with crossing fibers around cerebellar peduncles (CP) and MCP.

**Table 2:** Linear regression model comparing the FBA metrics in HIV+ and HIV– individuals, with age and sex included as covariates.

**ROI**	**HIV+**	**HIV–**	**Estimate**	**Std error**	***p*-value**	**Effect size**
**FD**						
Right PLIC	0.601	0.614	−0.020	0.006	**0.03**	0.46
Left PLIC	0.610	0.619	−0.013	0.007	0.28	0.29
Left SCR	0.352	0.356	−0.004	0.003	0.70	0.27
Right SCR	0.352	0.355	−0.001	0.004	0.81	0.17
Right ICP	0.402	0.406	0.007	0.012	0.81	0.08
Left ICP	0.427	0.427	0.012	0.011	0.72	0.01
MCP	0.448	0.443	0.004	0.009	0.81	0.14
**logFC**						
Right PLIC	−0.032	−0.007	−0.069	0.019	**<0.001**	0.30
Left PLIC	−0.028	−0.011	−0.067	0.019	**<0.001**	0.19
Left SCR	0.025	0.023	−0.043	0.019	**0.03**	0.03
Right SCR	0.026	0.029	−0.046	0.019	**0.03**	0.04
Right ICP	−0.017	0.017	−0.077	0.017	**<0.001**	0.43
Left ICP	−0.013	0.024	−0.075	0.016	**<0.001**	0.46
MCP	0.068	0.110	−0.094	0.021	**<0.001**	0.40
**FDC**						
Right PLIC	0.585	0.617	−0.065	0.015	**<0.001**	0.45
Left PLIC	0.599	0.621	−0.059	0.015	**<0.001**	0.32
Left SCR	0.361	0.367	−0.023	0.009	**0.02**	0.15
Right SCR	0.362	0.368	−0.020	0.010	0.06	0.16
Right ICP	0.396	0.415	−0.022	0.129	0.11	0.36
Left ICP	0.420	0.434	−0.019	0.012	0.12	0.27
MCP	0.485	0.502	−0.043	0.016	**0.02**	0.24

*Estimate is average difference between HIV+ and HIV– for which HIV– is taken as the reference group (two-tailed t-test, FDR corrected at the α = 0.05 significance level). PLIC, posterior limb of internal capsule; SCR, superior corona radiate; ICP, inferior cerebellar peduncle; MCP, middle cerebellar peduncle. Significant results are shown as bold. p-values are false discovery rate (FDR)-corrected*.

#### Region of Interest Analysis

Table 2 lists the mean and standard errors for several ROIs between the participants for the FBA metrics FD, logFC, and FDC for HIV+ and HIV– individuals. Linear models were further implemented to evaluate the relationship between fixel-based metrics and cohorts, including age and sex as covariates. We found a significant reduction in several ROIs in FBA metrics in HIV+ than in HIV– individuals. Linear regression models comparing DTI and fwcDTI metrics between HIV+ and HIV– cohorts are provided in Supplementary Table S1. None of the ROIs show any significant differences between the cohorts for the DTI and fwcDTI metrics.

**Figure 4:**
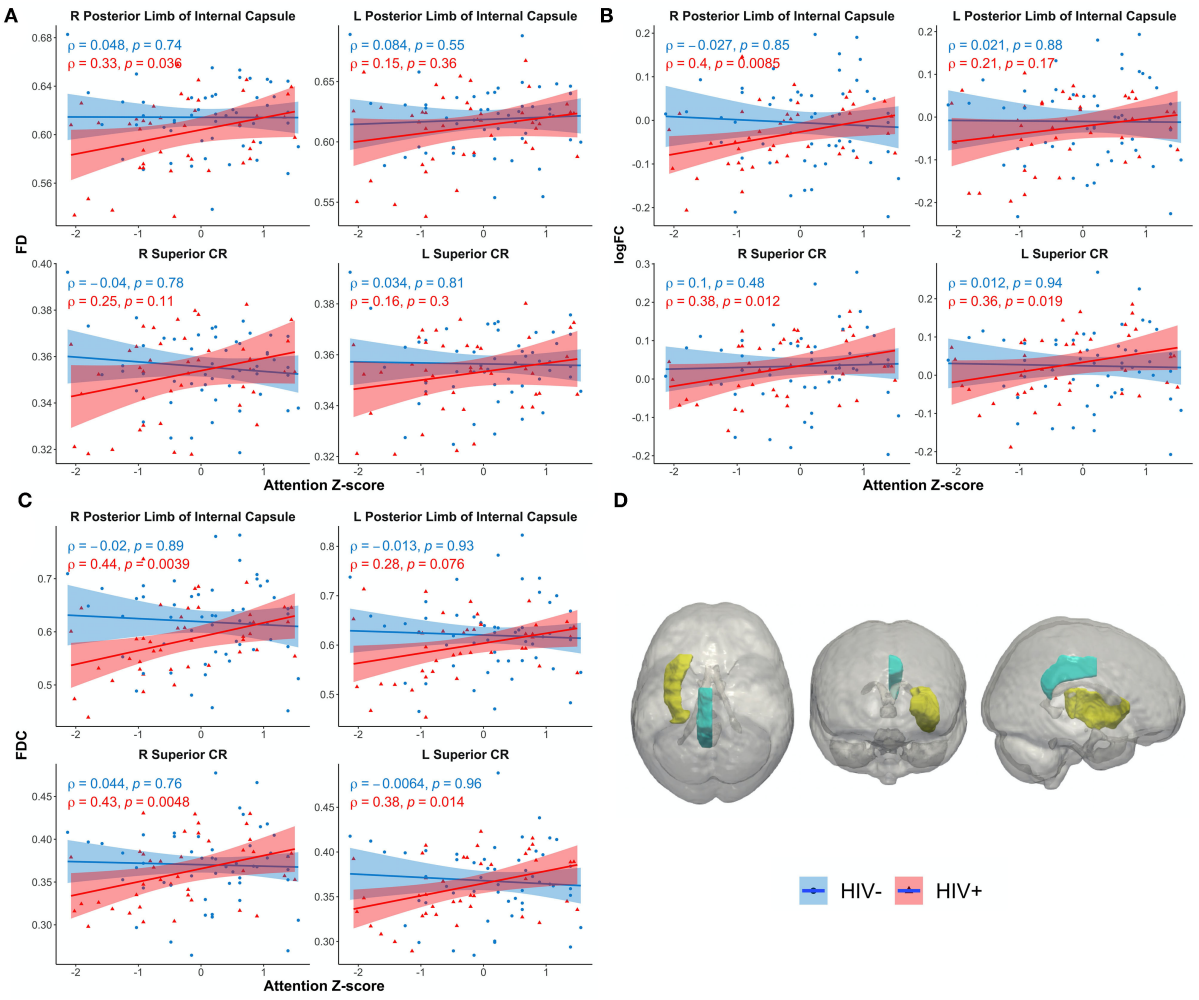
Scatterplots show attention *z*-score as a function of FBA metrics: **(A)** fiber density (FD), **(B)** log of fiber cross section (logFC), **(C)** fiber density and cross section (FDC), **(D)** JHU white matter atlas and corresponding regions of interest. Only significant regions are shown. Solid lines represent linear fit, and shaded areas represent the 95% confidence interval. IC, internal capsule (yellow); CR, corona radiata (teal); CR, corona radiata.

Figures 4A–C shows scatterplots examining the relationship between the attention domain *z*-scores and FBA metrics (FD, logFC, and FDC) for the right and left of PLIC and SCR. For HIV+ individuals, the right PLIC was found to be significant for FD (ρ = 0.33, *p* = 0.036), FDC (ρ = 0.44, *p* = 0.0039), and logFC (ρ = 0.4, *p* = 0.0085) while the right and left SCR were found to be significant in FDC (ρ = 0.43, *p* = 0.0048, and ρ = 0.38, *p* = 0.014, respectively). On the other hand, none of the ROIs for any metrics were significantly correlated with attention *z*-scores in the HIV– individuals. Figure 4D illustrates the corresponding ROIs.

Additionally, the right PLIC was also found to be significantly correlated with tau protein in HIV+ individuals (ρ = 0.32, *p* = 0.043; Supplementary Figure S2). However, no significant associations were observed between FBA metrics and NfL.

### Tract-Based Spatial Statistics (DTI and fwcDTI)

No significant differences between HIV+ and HIV– cohorts were observed in FA and FA_T_ using TBSS (Supplementary Figure S3). TBSS of FA_T_, while not significant, highlighted areas that were decreased in HIV+ individuals compared to HIV– individuals. It should be noted that in our study, the TBSS figures (Supplementary Figure S3) report regions found for *p* < 0.5. None of the regions survived for significance thresholding for *p* < 0.05.

### Machine Learning Classification

**Figure 5:**
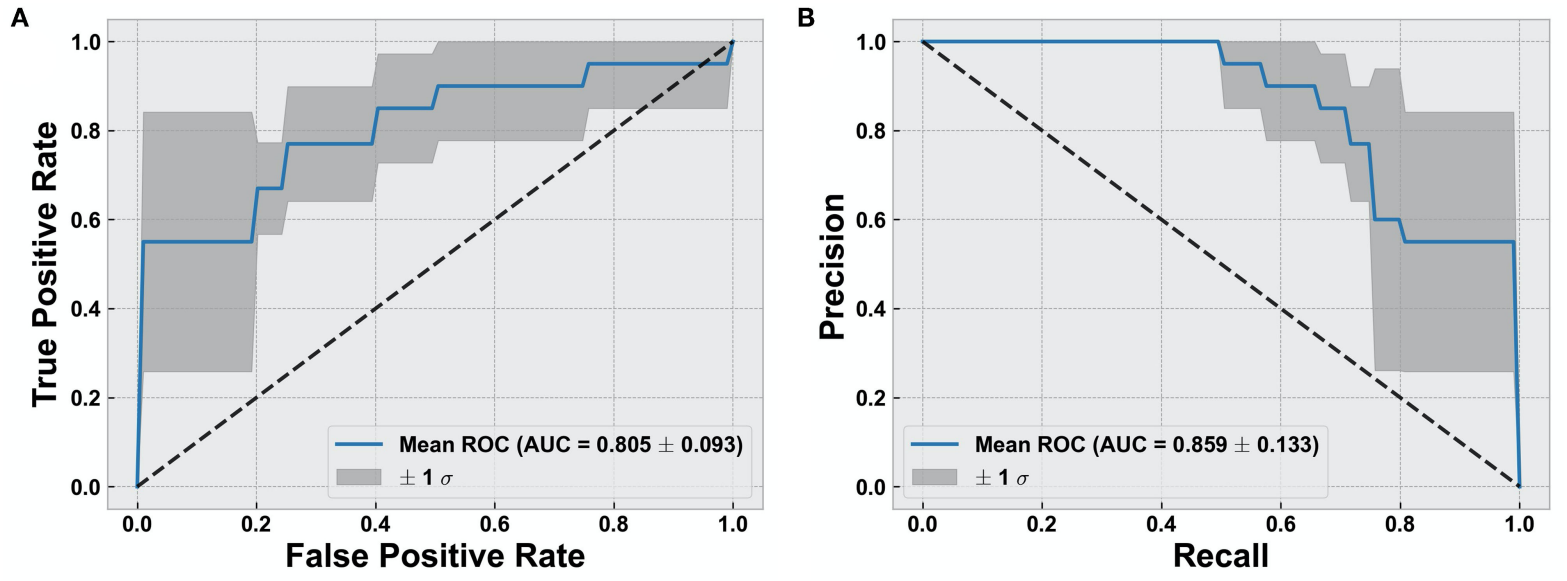
Evaluation of classification results using AdaBoost classifier. **(A)** Receiver operating characteristic (ROC) curve for cognitively normal compared to cognitively impaired (CI). **(B)** Precision vs. recall curve (PRC) for cognitively normal compared to CI. Solid line represents the mean curve using five-fold cross-validation. Shaded areas represent ± 1 standard deviation. AUC reported as mean ± standard deviation across five-fold.

Overall, we found that the use of fixel-based metrics resulted in a higher precision and recall compared to when using fwcDTI metrics. The adaptive boosting (AdaBoost) and random forest methods resulted in the highest recall and f1-score, and the highest precision was achieved with AdaBoost. Figure 5 shows the ROC and PRC curves using an AdaBoost multiclass classifier. Table 3 represents the sample averaged precision, recall, and f1-scores for other classifiers contrasting specificity of FBA and fwcDTI using five-fold cross-validation. In addition, the ROC and PRC curves for LDA, random forest, and naïve Bayes are provided in the Supplementary Figure S4. Decision boundaries for each classifier are also provided in Supplementary Figure S5.

**Table 3:** Precision, recall, and F1-scores for five different classifiers for FBA and fwcDTI metrics reported using five-fold cross-validation in HIV+ subjects only.

**Classifiers**	**Metrics**	**Precision**	**Recall**	***F*1-score**
LDA	FBA	0.62 (0.067)	0.60 (0.054)	0.58 (0.054)
	fwcDTI	0.50 (0.045)	0.43 (0.049)	0.46 (0.049)
Random forest	FBA	0.62 (0.076)	0.77 (0.045)	0.76 (0.049)
	fwcDTI	0.53 (0.040)	0.53 (0.045)	0.53 (0.045)
naïve Bayes	FBA	0.62 (0.067)	0.58 (0.044)	0.55 (0.045)
	fwcDTI	0.38 (0.045)	0.38 (0.049)	0.38 (0.045)
AdaBoost	FBA	**0.80 (0.049)**	**0.77 (0.045)**	**0.76 (0.049)**
	fwcDTI	0.48 (0.058)	0.46 (0.049)	0.47 (0.049)

*Scores reported as weighted average across three classes. FBA, fixel-based analysis; fwcDTI, free water corrected diffusion tensor imaging; LDA, linear discriminant analysis. Values are reported as mean (SE). Highest performance is shown as bold*.

## Discussion

In this study, we evaluated fiber tract-specific WM changes in HIV infection using FBA, DTI, and fwcDTI metrics. The major findings of this work were as follows: (a) HIV+ individuals exhibit axonal degradation within the PLIC, CP, and SCR as revealed by FBA. (b) Similar trends were observed using TBSS of FA and FA_T_. In contrast to FA, FA_T_ showed trends toward more areas that were decreased in HIV+ individuals compared to HIV– individuals. (c) FBA metrics in PLIC and SCR exhibit significant positive associations with attention cognitive *z*-scores in HIV+ individuals. (d) Machine learning classifiers for FBA reliably distinguished between cognitively normal patients and those with cognitive impairment in patients with HIV infection.

To the best of our knowledge, this is the first study investigating FBA and TBSS of fwcDTI metrics (FA_T_ and MD_T_) in HIV-infected individuals. The work presented here provides a comprehensive and robust framework for evaluating brain injury (and secondary chronic inflammation) in the setting of HIV. Chronic neuroinflammation results in damage to the CNS, alteration of the blood–brain barrier (BBB), and chronic edema (65). Changes in whole-brain FBA were found along distinct fiber tracts associated with motor and attention cognitive domains. In particular PLIC, CP, MCP, and SCR were affected and exhibited reduced fiber density and fiber bundle cross section in HIV+ individuals compared to HIV– individuals. ROI-based analysis revealed lower mean fixel-based metrics in the HIV+ cohort compared to the HIV– cohort, consistent with those obtained from the whole-brain FBA results.

Previous work using DTI has shown that FA is decreased in corticospinal tract and that MD is increased in the corticospinal tract (CST) bilaterally (23). However, FW contamination results in fitting each voxel with an isotropic tensor, leading to an erroneous conclusion that FA is decreased in the presence of edema (12). Consistent with previous work, our findings suggest that axonal degeneration occurs only in fixels associated with the CST in HIV infection, but accounting for edema and FW contamination. Moreover, FBA is a more robust method to evaluate WM structural integrity compared to TBSS, with or without FW correction. This is likely because constrained spherical deconvolution is dependent on the response function, which is estimated separately and independently for each tissue type, ultimately better modeling the FOD (48).

In addition, our findings are consistent with previous work investigating WM in HIV infection, and with the clinical presentation of HAND (2). However, the present study emphasizes FBA to provide a more robust means to evaluate WM structural integrity independent of partial volume effects and FW contamination. Although we only saw trends using TBSS of fwcDTI, it is reasonable to implement this approach to DTI data. Clinically, HAND is a spectrum of disorders in which patients may present with difficulties in cognition, particularly declines in psychomotor processing, attention, and memory. Of interest, the role of the corona radiata in motor pathways is well-established; however, recent studies have suggested that the corona radiata is related to attention as well (66). As a major WM intersection, it is possible that damage to the corona radiata, observed in this study, affects both corticospinal fibers as well as association fibers passing through the SCR, contributing to the diffuse cognitive changes seen in HAND, particularly psychomotor slowing. Additionally, the default mode network (DMN) has been implicated in HIV and HAND (9). The DMN is primarily composed of the medial prefrontal cortex, posterior cingulate cortex, precuneus, and angular gyrus. Moreover, the DMN, a task-negative network, is associated with attention and memory. Thus, it might be possible that degeneration of association fibers passing through the corona radiata and internal capsule disrupt connections to the DMN (67).

We also investigated the relationship between fixel-based metrics (i.e., FD, logFC, and FDC) and inflammatory blood markers including CD4, VL, and neuronal markers NfL, and tau protein in HIV+ individuals. The PLIC was the only structure significantly correlated with tau protein (ρ = 0.32, *p* = 0.043). No other blood markers were significantly correlated with fixel-based metrics. Tau protein is a component of the neurofibrillary tangles most often associated with Alzheimer's disease (68). However, increasing evidence suggests that chronic neuroinflammation in the setting of HIV infection predisposes HIV+ individuals to premature neurodegeneration as measured by tau protein (69).

Lastly, four machine learning classifiers were used to classify cognitive status in HIV+ individuals using fixel-based and free water corrected DTI metrics. In general, we observed that fixel-based metrics results in improved performance as measured with precision, recall, and f1-score, compared to fwcDTI metrics. Additionally, ensemble machine learning methods (random forest and AdaBoost) resulted in higher precision, recall and f1-score as compared with discriminant methods (LDA and naïve Bayes). This is likely because ensemble methods utilize bootstrapping and bagging methods that lower the variance of the overall model. However, as suggested by the decision boundaries in Supplementary Figure S5, it is possible that these findings are due to overfitting given the small sample size, and should be interpreted with caution. Additionally, we observed that AdaBoost resulted in a higher precision than the random forest classifier, likely because in AdaBoost, weak classifiers are built sequentially propagating the errors from prior weak learners, whereas in random forest algorithms the decision trees are grown in parallel and as such are independent from each other. Inclusion of other relevant imaging metrics and biomarkers is likely to further improve prediction of developing HAND.

This study has some limitations. First, only one of the HIV+ subjects in the study had mild neurological disorder (MND); therefore, our study was mostly composed of cognitively normal subjects and patients with ANI. However, MND and ANI were combined and categorized as CI. Second, the proportion of male and female subjects was not equal in the HIV+ cohort. However, FBA and DTI metrics were not significantly different in males vs. females in our HIV– participants, which had a more equal representation. Though sex was used as a covariate in between-group analyses, we cannot rule out this possible bias in our findings. Third, as noted previously, the FD is dependent on the *b*-value, and a higher correspondence between the intra-axonal volume occurs at higher *b*-values (*b* >2,000 s/mm^2^). Thus, it is important to note that at *b* = 1,000 s/mm^2^, the AFD may reflect the overall WM density and not just the intra-axonal volume fraction (44). Fourth, it is worth noting that given the small size of our dataset, classification results obtained here are preliminary and need to be further validated in a larger dataset. However, utilization of stratified sampling, standardization, cross-validation, and boosting algorithms have been shown to obviate overfitting when dealing with very small datasets (70, 71). Future research will investigate the utility of fixel-based metrics in evaluating HIV-associated neuroinflammation longitudinally and developing prognostic machine learning models for predicting HAND longitudinally.

## Conclusions

Our findings suggest that FBA may be reflective of WM structural integrity in the setting of chronic neuroinflammation in HIV population. Our results indicate that degeneration occurs along specific fiber tracts, which manifests as both macrostructural and microstructural alterations, particularly in the internal capsule and corona radiata in HIV+ individuals. Moreover, our findings are consistent with the clinical presentation of HAND, which often presents as psychomotor slowing with impaired attention, memory, and fine motor function. TBSS-based analysis of free water corrected and uncorrected DTI metrics showed decreasing trends between the HIV+ and HIV– control group. However, these were not significant, suggesting lower sensitivity for the level of pathology in the cohort under investigation compared to FBA. Therefore, FBA may provide a sensitive biomarker to monitor axonal degeneration in individuals with HIV infection.

## Data Availability Statement

The data analyzed in this study is subject to the following licenses/restrictions: Anonymized data will be made available on reasonable request, pending appropriate institutional review board approvals. Requests to access these datasets should be directed to Giovanni Schifitto, Giovanni_Schifitto@URMC.Rochester.edu.

## Ethics Statement

The studies involving human participants were reviewed and approved by the University of Rochester Research Subjects Review Board (RSRB). The patients/participants provided their written informed consent to participate in this study.

## Author Contributions

AFi: study concept, image processing, data analysis and interpretation, manuscript writing, and original draft. AFa: image processing, data analysis and interpretation, and manuscript review for intellectual content. MW: cognitive data collection and manuscript review for intellectual content. XQ: statistical interpretation and manuscript review for intellectual content. MU: study concept and interpretation and manuscript writing and review for intellectual content. JZ: manuscript review for intellectual content. GS: project administration, funding acquisition, interpretation, and manuscript review for intellectual content. All authors contributed to the article and approved the submitted version.

## Funding

This work was supported by the National Institutes of Health (NIH) Grants R01-MH099921, R01-AG054328, and R01-MH118020.

## Conflict of Interest

The authors declare that the research was conducted in the absence of any commercial or financial relationships that could be construed as a potential conflict of interest.

## Publisher's Note

All claims expressed in this article are solely those of the authors and do not necessarily represent those of their affiliated organizations, or those of the publisher, the editors and the reviewers. Any product that may be evaluated in this article, or claim that may be made by its manufacturer, is not guaranteed or endorsed by the publisher.

## Supplementary Material

Supplementary Figure S1 Boxplots of cognitive domain *Z*-scores by HIV-status. Worse scores (negative *Z*-scores) for attention, memory, and overall cognitive summary score were observed in the HIV+ individuals compared to HIV uninfected individuals. Significant group differences are shown with *p* < 0.05.

Supplementary Figure S2 Relationship between fiber density and cross-section (FDC) and Tau protein in HIV+ cohort. Only significant regions shown. Solid lines represent linear fit, and shaded areas represent the 95% confidence interval. IC, internal capsule.

Supplementary Figure S3 Group comparisons of **(A)** FA, **(B)** FA_T_, and **(C)** MD_T_ using TBSS. Statistical maps thresholded at 0.5 (not significant) show regions where FA and FA_T_ are reduced in HIV+ individuals compared to HIV– individuals. FA, fractional anisotropy; FA_T_, free water corrected fractional anisotropy; Blue represents reduced FA or FA_T_ in the HIV+ compared to HIV– individuals.

Supplementary Figure S4 Evaluation of classification results using **(A)** LDA, **(B)** random forest, and **(C)** naïve Bayes. Left column: receiver operating characteristic (ROC) curve for cognitively normal compared to cognitively impaired (CI). Right column: precision-recall curve (PRC) for cognitively normal compared to CI. Solid line represents the mean curve using five-fold cross validation. Shaded areas represent ± 1 standard deviation. AUC reported as mean ± standard deviation across five-folds.

Supplementary Figure S5 Decision boundaries of classifiers **(A)** LDA, **(B)** Naïve Bayes, **(C)** Random Forest, and **(D)** AdaBoost. Shaded red area indicates region where instances are classified as cognitively normal, and gray region indicates the area where instances are classified as cognitively impaired (CI). The border between these two regions represents the decision boundary for each classifier. PC1: first principal component, PC2: second principal component.

## Abbreviations

cART: combined antiretroviral therapy
CNS: central nervous system
BBB: blood–brain barrier
WM: white matter
HAND: HIV-associated neurocognitive disorder
FW: free water
DTI: diffusion tensor imaging
FA: fractional anisotropy
AD: axial diffusivity
RD: radial diffusivity
MD: mean diffusivity
TBSS: tract-based spatial statistics
CSF: cerebrospinal fluid
FA_T_: free water corrected FA
MD_T_: free water corrected MD
FBA: fixel-based analysis
FD: fiber density
FC: fiber bundle cross section
FDC: fiber density cross section
AFD: apparent fiber density
FOD: fiber orientation distribution
MT-CSD: multi tissue constrained spherical deconvolution
GM: gray matter
NfL: neurofilament light chain
VVL: viral load
GLM: general linear model
CFE: connectivity-based fixel enhancement
FDR: false discovery rate
ROI: region of interest
TFCE: threshold-free cluster enhancement
WNL: within normal limits
ANI: asymptomatic neurological impairment
MND: minor neurocognitive impairment
CI: cognitively impaired
kPCA: kernel principal component analysis
LDA: linear discriminant analysis
PPV: positive predictive value
ROC: receiver operating characteristic
AUC: area under the curve
fwcDTI: free water corrected DTI
DWI: diffusion weighted imaging
PLIC: posterior limb of the internal capsule
MCP: middle cerebellar peduncles
SCR: superior corona radiata
CP: cerebellar peduncle
SLF: superior longitudinal fasciculus
AdaBoost: adaptive boosting
PRC: precision-recall curve
DMN, default mode network, CST: corticospinal tract.
