# Supplementary figures and images for: Fixel-Based Analysis and Free Water Corrected DTI Evaluation of HIV-Associated Neurocognitive Disorders
Source: Front Neurol. 2021 Nov 4;12:725059. doi: 10.3389/fneur.2021.725059 (PMC8600320; doi:10.3389/fneur.2021.725059)

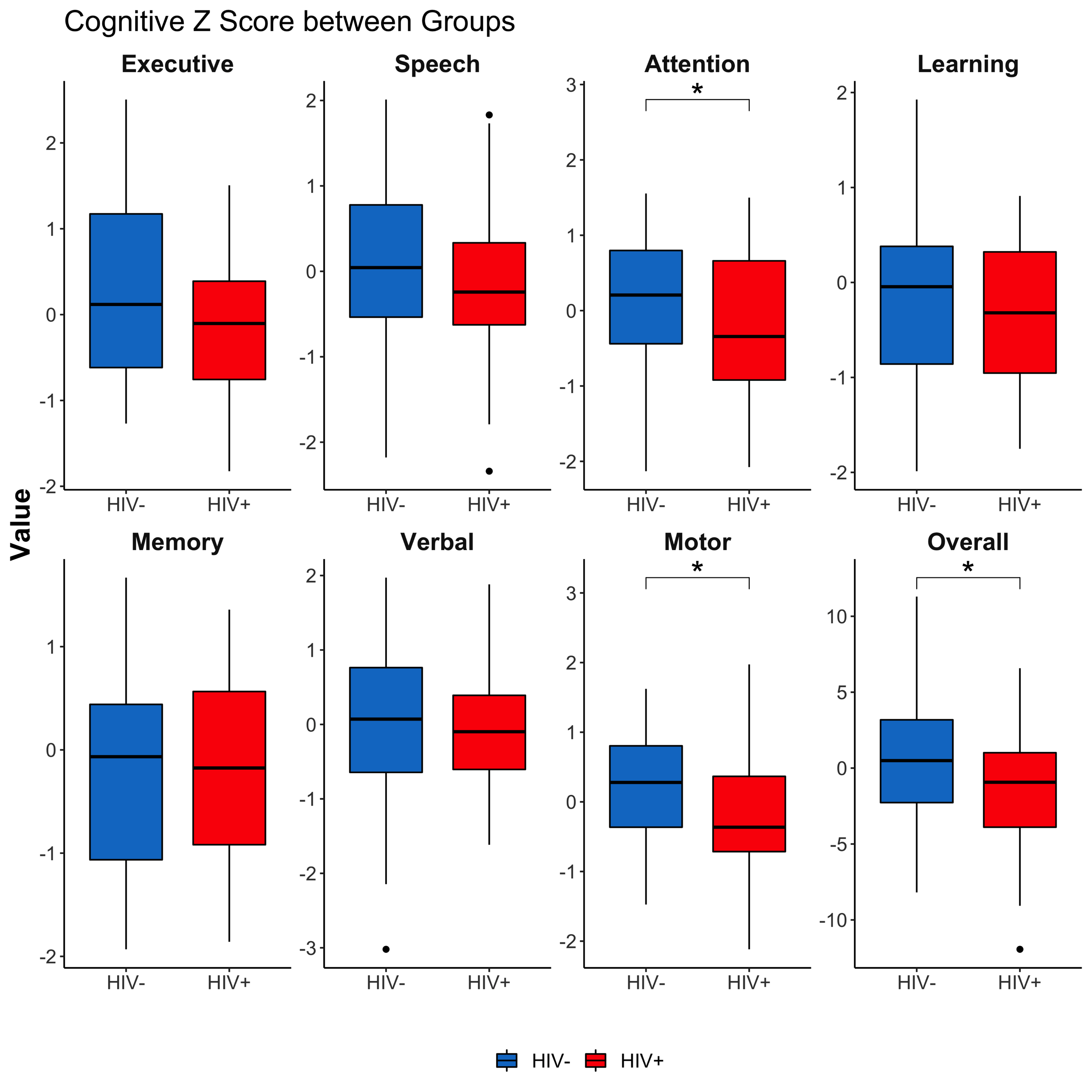

Supplement: Supplementary Figure S1 — Boxplots of cognitive domain Z-scores by HIV-status. Worse scores (negative Z-scores) for attention, memory, and overall cognitive summary score were observed in the HIV+ individuals compared to HIV uninfected individuals. Significant group differences are shown with p < 0.05. [file Image_1.tiff]

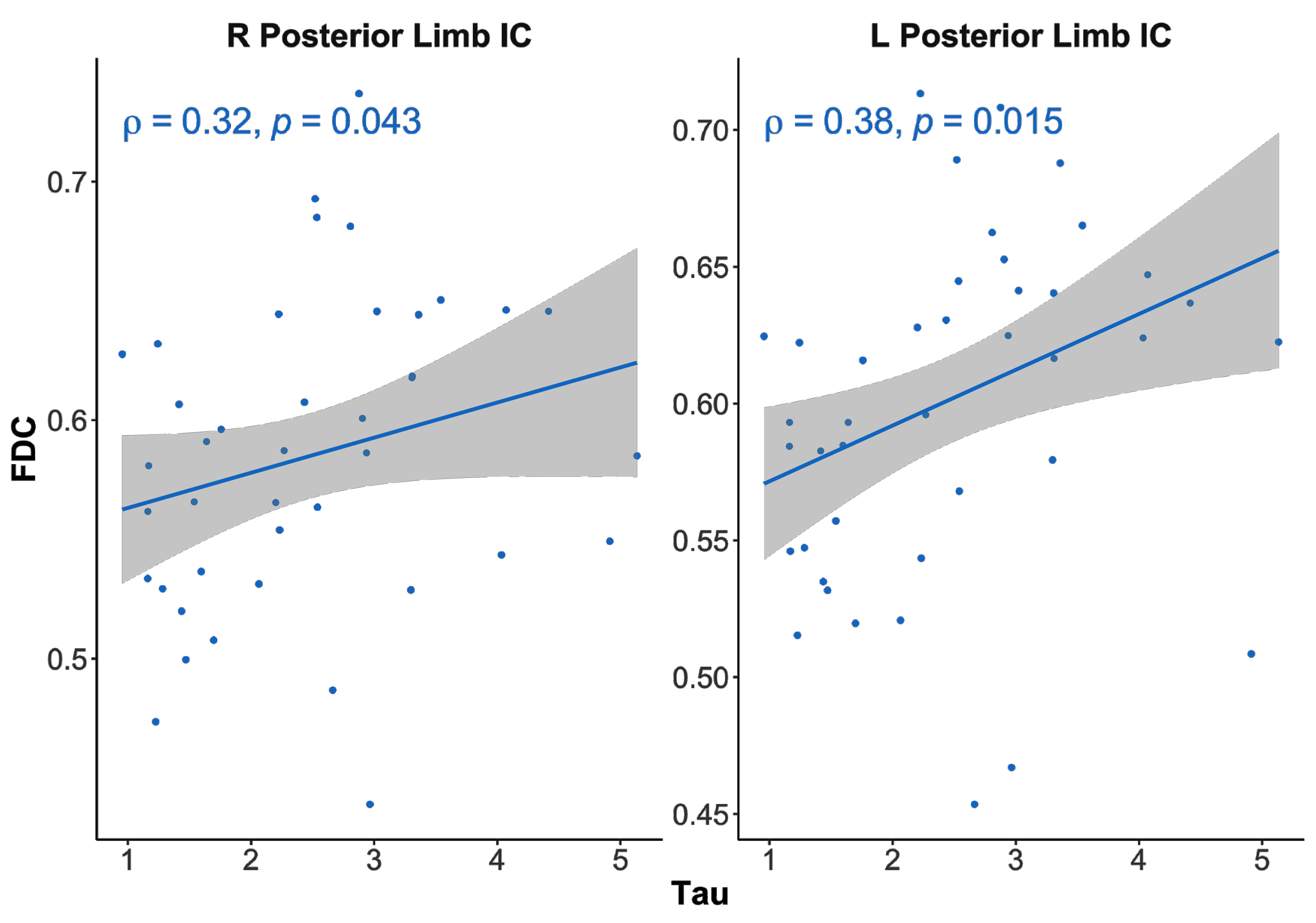

Supplement: Supplementary Figure S2 — Relationship between fiber density and cross-section (FDC) and Tau protein in HIV+ cohort. Only significant regions shown. Solid lines represent linear fit, and shaded areas represent the 95% confidence interval. IC, internal capsule. [file Image_2.tiff]

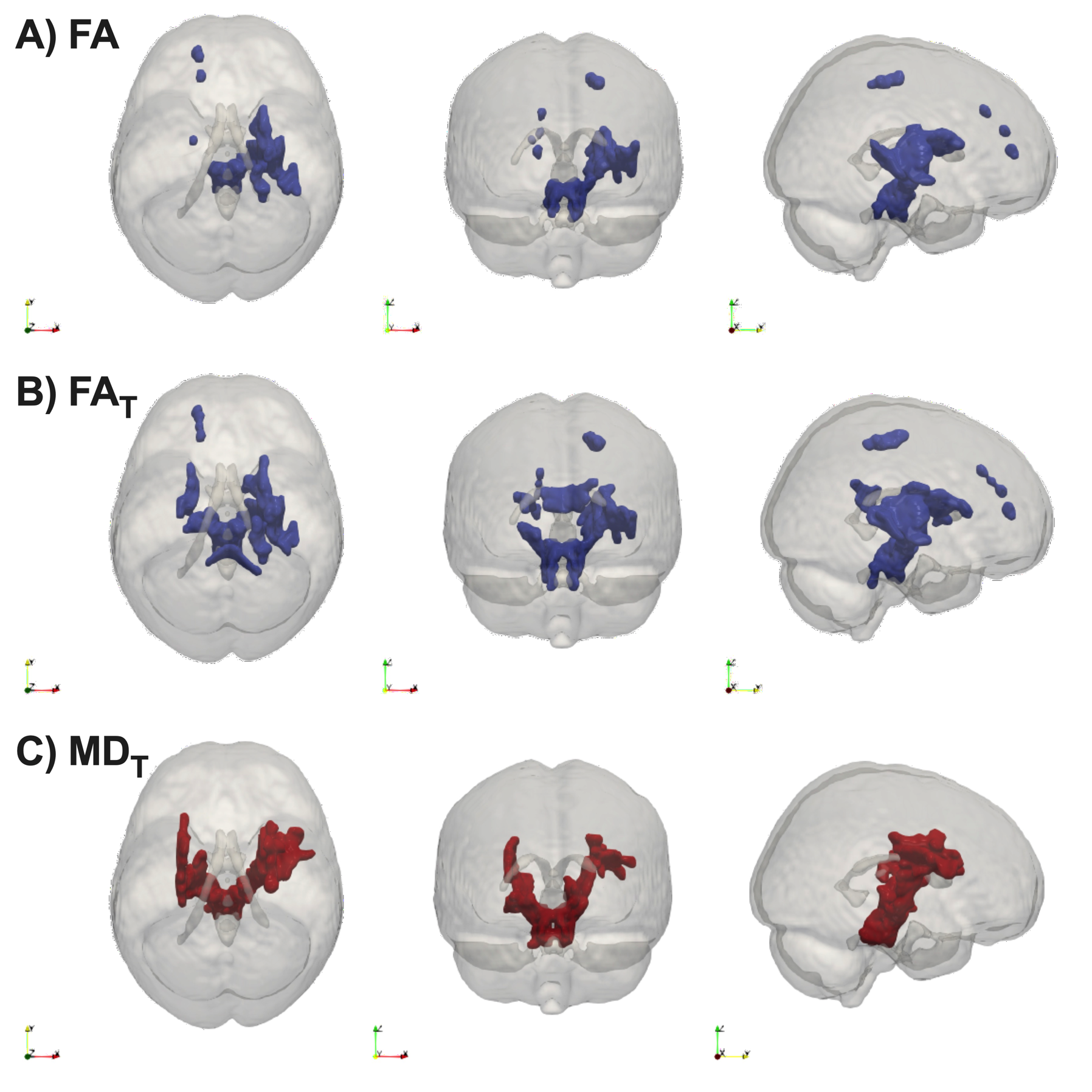

Supplement: Supplementary Figure S3 — Group comparisons of (A) FA, (B) FAT, and (C) MDT using TBSS. Statistical maps thresholded at 0.5 (not significant) show regions where FA and FAT are reduced in HIV+ individuals compared to HIV– individuals. FA, fractional anisotropy; FAT, free water corrected fractional anisotropy; Blue represents reduced FA or FAT in the HIV+ compared to HIV– individuals. [file Image_3.tiff]

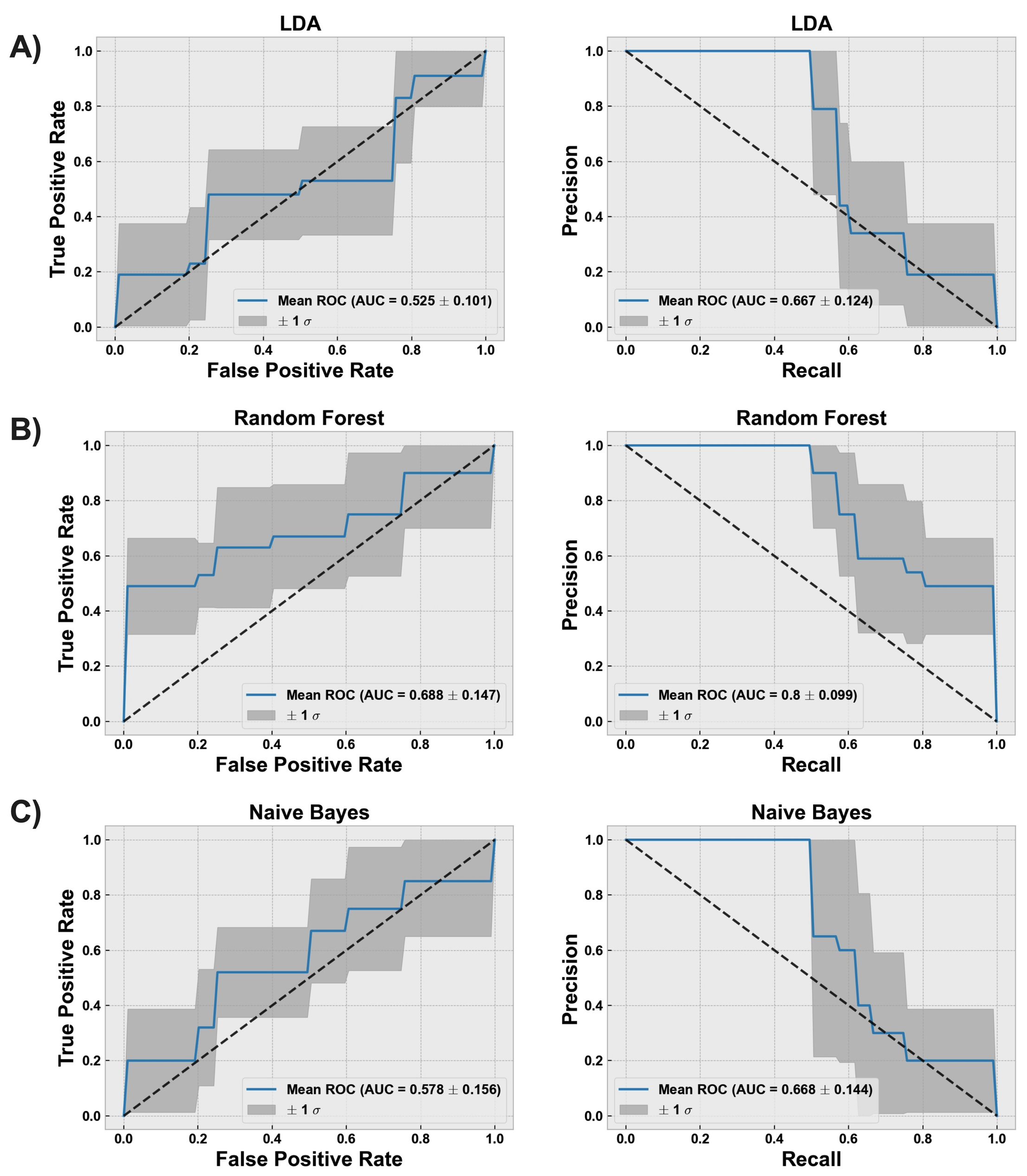

Supplement: Supplementary Figure S4 — Evaluation of classification results using (A) LDA, (B) random forest, and (C) naïve Bayes. Left column: receiver operating characteristic (ROC) curve for cognitively normal compared to cognitively impaired (CI). Right column: precision-recall curve (PRC) for cognitively normal compared to CI. Solid line represents the mean curve using five-fold cross validation. Shaded areas represent ± 1 standard deviation. AUC reported as mean ± standard deviation across five-folds. [file Image_4.tiff]

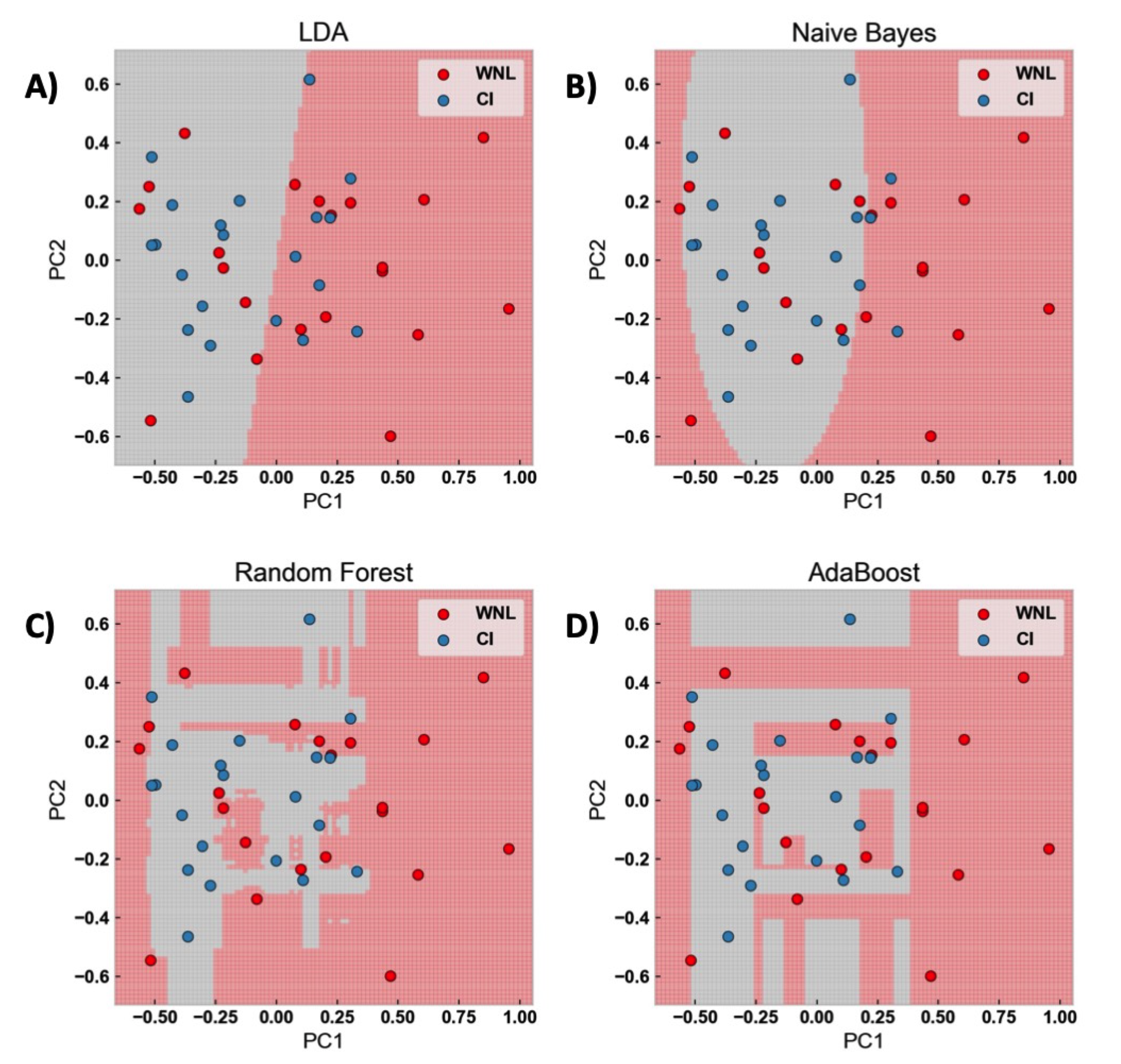

Supplement: Supplementary Figure S5 — Decision boundaries of classifiers (A) LDA, (B) Naïve Bayes, (C) Random Forest, and (D) AdaBoost. Shaded red area indicates region where instances are classified as cognitively normal, and gray region indicates the area where instances are classified as cognitively impaired (CI). The border between these two regions represents the decision boundary for each classifier. PC1: first principal component, PC2: second principal component. [file Image_5.tiff]
