# Supplementary material for: Fixel-Based Analysis and Free Water Corrected DTI Evaluation of HIV-Associated Neurocognitive Disorders
Source: Front Neurol. 2021 Nov 4;12:725059. doi: 10.3389/fneur.2021.725059 (PMC8600320; doi:10.3389/fneur.2021.725059)
Supplement: Supplementary file 6 [file Table_1.docx]

**Supplementary Table S1.** Linear regression model comparing the DTI and fwcDTI metrics in HIV+ and HIV- individuals, with age and sex included as covariates.

| **ROI** | **HIV+** | **HIV-** | **Estimate** | **Std Error** | ***p-value*** | **Effect Size** |
| --- | --- | --- | --- | --- | --- | --- |
| **FA** |  |  |  |  |  |  |
| Right PLIC | 0.590 | 0.592 | -0.0054 | 0.0046 | 0.608 | 0.106 |
| Left PLIC | 0.585 | 0.585 | -0.0038 | 0.0049 | 0.608 | 0.021 |
| Left SCR | 0.442 | 0.439 | 0.0044 | 0.0049 | 0.608 | 0.144 |
| Right SCR | 0.442 | 0.439 | 0.0026 | 0.0049 | 0.608 | 0.134 |
| Right ICP | 0.394 | 0.403 | -0.0139 | 0.0080 | 0.597 | 0.266 |
| Left ICP | 0.381 | 0.386 | -0.0098 | 0.0091 | 0.608 | 0.119 |
| MCP | 0.420 | 0.413 | 0.0038 | 0.0065 | 0.608 | 0.257 |
| **MD** |  |  |  |  |  |  |
| Right PLIC | 7.00e-4 | 7.00e-4 | 3.30e-6 | 3.70e-6 | 0.590 | 0.231 |
| Left PLIC | 7.10e-4 | 7.00e-4 | 1.50e-6 | 3.70e-6 | 0.801 | 0.208 |
| Left SCR | 7.30e-4 | 7.20e-4 | 4.80e-6 | 6.00e-6 | 0.590 | 0.264 |
| Right SCR | 7.10e-4 | 7.10e-4 | 5.90e-6 | 5.90e-6 | 0.590 | 0.261 |
| Right ICP | 8.30e-4 | 8.20e-4 | 2.62e-5 | 1.48e-5 | 0.562 | 0.259 |
| Left ICP | 8.30e-4 | 8.30e-4 | 5.99e-5 | 1.41e-5 | 0.971 | 0.099 |
| MCP | 8.10e-4 | 8.20e-4 | -1.20e-5 | 1.23e-5 | 0.590 | 0.077 |
| **FA_T_** |  |  |  |  |  |  |
| Right PLIC | 0.593 | 0.596 | -0.0061 | 0.0048 | 0.357 | 0.152 |
| Left PLIC | 0.588 | 0.589 | -0.0048 | 0.0052 | 0.502 | 0.040 |
| Left SCR | 0.457 | 0.454 | 0.0010 | 0.0052 | 0.843 | 0.131 |
| Right SCR | 0.454 | 0.452 | 0.0030 | 0.0053 | 0.663 | 0.106 |
| Right ICP | 0.448 | 0.458 | -0.0156 | 0.0073 | 0.246 | 0.293 |
| Left ICP | 0.448 | 0.452 | -0.0099 | 0.0076 | 0.357 | 0.122 |
| MCP | 0.496 | 0.496 | -0.0085 | 0.0054 | 0.357 | 0.013 |
| **MD_T_** |  |  |  |  |  |  |
| Right PLIC | 7.00e-4 | 7.00e-4 | 4.00e-6 | 3.80e-6 | 0.698 | 0.288 |
| Left PLIC | 7.10e-4 | 7.00e-4 | 2.00e-6 | 3.70e-6 | 0.698 | 0.238 |
| Left SCR | 7.20e-4 | 7.20e-4 | 3.60e-6 | 5.60e-6 | 0.698 | 0.260 |
| Right SCR | 7.10e-4 | 7.10e-4 | 5.80e-6 | 5.70e-6 | 0.698 | 0.269 |
| Right ICP | 7.70e-4 | 7.70e-4 | 6.20e-6 | 1.17e-5 | 0.698 | 0.017 |
| Left ICP | 7.80e-4 | 7.90e-4 | 6.40e-6 | 2.89e-5 | 0.826 | 0.063 |
| MCP | 7.30e-4 | 7.60e-4 | -3.94e-5 | 1.64e-5 | 0.130 | 0.408 |

Note: Estimate is average difference between HIV+ and HIV- for which HIV- is taken as the reference group (two-tailed t-test, FDR corrected at the α = 0.05 significance level). PLIC: posterior limb of Internal Capsule, SCR: Superior Corona Radiata, ICP: inferior cerebellar peduncle MCP: Middle Cerebellar Peduncle, fwcDTI: free water corrected DTI.
